# Metalloantibiotic functionalized pHEMA for therapeutic contact lenses

**DOI:** 10.1039/d6ra04865j

**Published:** 2026-08-07

**Authors:** Sotiris K. Hadjikakou, Christina N. Banti

**Affiliations:** a Biological Inorganic Chemistry Laboratory, Department of Chemistry, University of Ioannina Ioannina 45110 Greece shadjika@uoi.gr cbanti@uoi.gr +30-26510-08362 +30-26510-08374

## Abstract

Ocular infections remain a major cause of visual impairment worldwide, while the low bioavailability of conventional eye drops limits therapeutic efficacy and patient compliance. Herein, we report the development of therapeutic contact lenses based on poly(2-hydroxyethyl methacrylate) (pHEMA) functionalized with the antibacterial silver(i) metalloantibiotics {[Ag(CipH)_2_]NO_3_·0.75MeOH·1.2H_2_O} (CipAg) and [Ag(pen)(CH_3_OH)]_2_ (PenAg), yielding the biomaterials pHEMA@CipAg and pHEMA@PenAg, respectively. Successful incorporation of the antimicrobial agents into the hydrogel matrix was confirmed by refractive index, X-ray fluorescence (XRF), solid-state UV-vis spectroscopy and differential scanning calorimetry (DTG/DSC). Among the tested materials, pHEMA@CipAg exhibited the highest antibacterial activity, reducing bacterial viability to 1.3%, 0.4% and 1.4% against *P. aeruginosa*, *S. epidermidis* and *S. aureus*, respectively. Moreover, pHEMA@CipAg generated large inhibition zones ranging from 30 to 41 mm and removed 66.7% and 77.6% of preformed *P. aeruginosa* and *S. aureus* biofilms, respectively. The metalloantibiotic-based lenses consistently outperformed the corresponding lenses containing the parent antibiotics, demonstrating the beneficial effect of silver(i) coordination on antimicrobial efficacy. Importantly, the materials displayed acceptable biocompatibility toward human corneal epithelial cells (HCEC), while micronucleus, *Artemia salina* and *Allium cepa* assays revealed no significant toxicological concerns.

## Abstract 2

Silver(i) metalloantibiotic-functionalized pHEMA contact lenses exhibit potent antibacterial and antibiofilm activity against ocular pathogens while maintaining acceptable biocompatibility.

## Graphical abstract

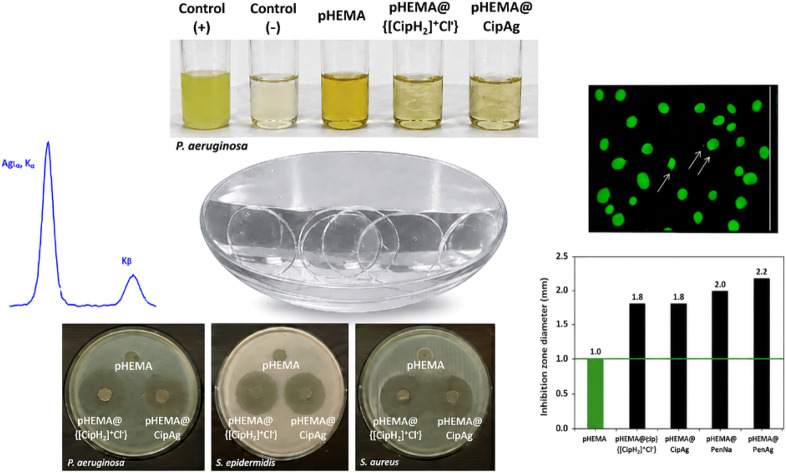

## Introduction

Ocular infections are a major public health concern worldwide and can cause visual impairment or even permanent vision loss.^1–3^ In addition to their clinical consequences, they impose a substantial socioeconomic burden due to healthcare costs, loss of productivity, and, in severe cases, hospitalization.^4–9^ Consequently, the development of effective therapeutic platforms with improved antimicrobial efficacy and ocular drug delivery such as contact lenses remains highly desirable.^10–13^

Current management strategies for the treatment of ocular infections rely primarily on antimicrobial therapy, supportive care, and, in severe cases, hospital-based interventions. Bacterial infections such as conjunctivitis and microbial keratitis are commonly treated with topical antibiotics, including fluoroquinolones and aminoglycosides, while systemic therapy and anti-inflammatory agents may be required in more severe cases.^1,3,7^ Eye drops account approximately 90% of therapy for common occular diseases.^12^ The bioavailability of topical drugs is less than 5% ^12^ so they required frequent and high-dose administrations to achieve the desired effect,^10^ as a result, increasing the potential for toxic side effects such as cellular damage, inflammation of the ocular surface, and leading to discomfort and poor patient compliance.^11^

Drug-loaded contact lenses, on the other hand, have been developed to improve the ocular drug delivery and bioavailability, without compromising ocular transparency.^10^ On February 25, 2022, Johnson announced that the FDA had approved its new therapeutic contact lens as the first drug-loading contact lens in the world.^12^ 2-Hydroxyethyl methacrylate (HEMA) is one of the most important contact lens hydrogel materials in use. HEMA can act as polymers with high water content and oxygen permeability; HEMA-derived hydrogels dissolved in water account for approximately 22% of the commercial contact lens market.^12^

**Scheme 1:**
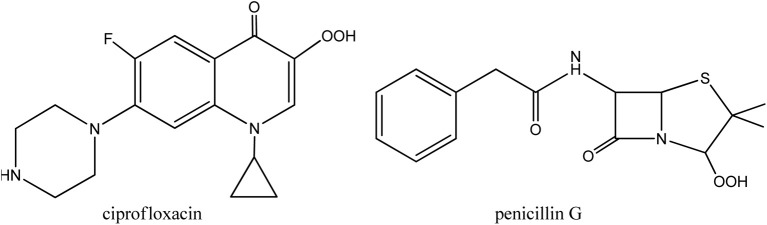
Formula of ciprofloxacin and penicillin G.

In the course of our study our research on developing innovative therapeutic contact lenses,^14–21^ we report the preparation and physicochemical characterization of p-hydroxyethyl-methacrylate (pHEMA) hydrogels functionalized by the known antibacterial metalloantibiotics {[Ag(cipH)_2_]NO_3_·0.75MeOH·1.2H_2_O} (CipAg) (cipH = ciprofloxacin, Scheme 1)^14^ and [Ag(pen)(CH_3_OH)]_2_ (PenAg) (PenH = penicillin G, Scheme 1),^17^ namely pHEMA@CipAg and pHEMA@PenAg, respectively. The hydrogels of pHEMA@{[CipH_2_]^+^Cl^−^} and pHEMA@PenNa were also syntethised for comparison reason. The materials pHEMA@CipAg and pHEMA@PenAg were characterized by refractive index, XRF, solid state UV-vis spectroscopies as well as DTG/DSC. The antimicrobial potential of the materials was assessed against Gram-negative bacterial strain (*P. aeruginosa*) and Gram-positive strains (*S. epidermidis* and *S. aureus*) by the means of bacterial viability, inhibition zone (IZ) and the ability of removal of preformed biofilm. The *in vitro* toxicity of the biomaterials was evaluated against normal human corneal epithelial cells (HCECs) whereas the *in vitro* genotoxicity was evaluated by the micronucleus (MN) assay towards HCECs. The *Artemia salina* and *Allium cepa* models were used for the evaluation of *in vivo* toxicity and genotoxicity of them.

## Results and discussion

### General aspects

**Scheme 2:**
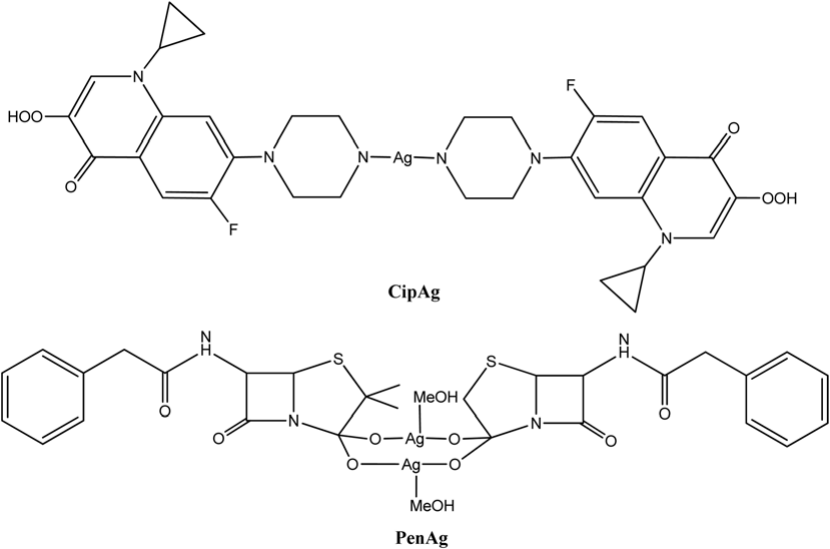
Structure of CipAg and PenAg.

The metalloantibiotics CipAg and PenAg (Scheme 2) were synthesized according to previously reported procedures.^14,17^ Briefly, CipAg, is obtained by the reacting silver nitrate with ciprofloxacin (1 : 2 molar ratio) in methanol and acetonitrile solution. The metalloantibiotic [Ag(pen)(CH_3_OH)]_2_ (PenAg) was synthesized by reacting silver nitrate with sodium penicillin G in 1 : 1 molar ratio in methanol/water solution.

**Scheme 3:**
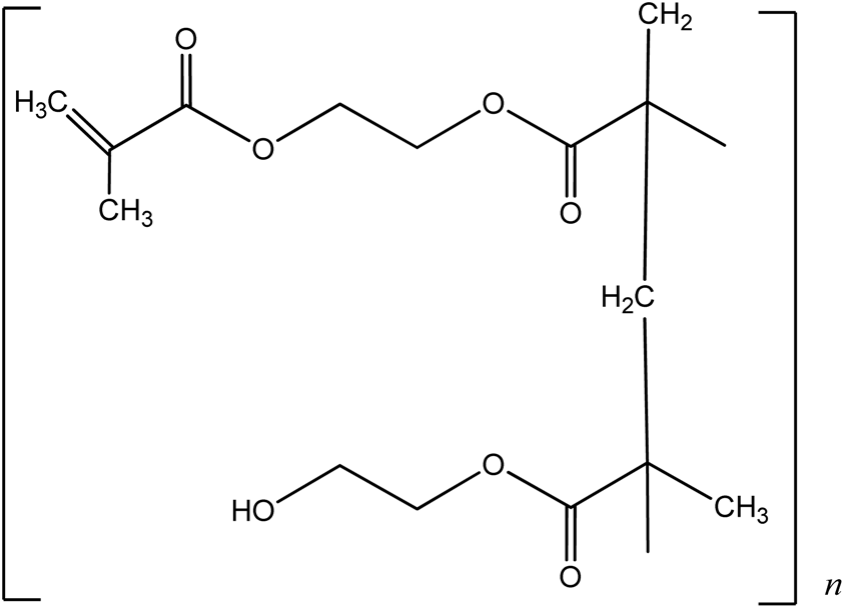
Structure of pHEMA.

Solutions of CipAg, {[CipH_2_]^+^Cl^−^}, PenAg, and PenNa (1 mM) were incorporated into pHEMA during the polymerization process (Scheme 3), leading to the formation of the biomaterials pHEMA@CipAg, pHEMA@{[CipH_2_]^+^Cl^−^}, pHEMA@PenAg, and pHEMA@PenNa. Their successful incorporation into the pHEMA matrix was qualitatively and quantitively confirmed by XRF, solid-state UV spectroscopy and DTG/DSC analyses. No oxygen permeability measurements were carried out since such measurements were beyond the scope of the present study and require specialized instrumentation that is not currently available to us.

### Refractive index

The refractive indices of pHEMA@CipAg, pHEMA@{[CipH_2_]^+^Cl^−^}, pHEMA@PenAg, and pHEMA@PenNa were determined after equilibration in saline solution. The measured refractive indices were 1.4345, 1.4350, 1.4341, and 1.4340, respectively, values that are essentially identical to that of free pHEMA (1.434), indicating that incorporation of the antimicrobial agents does not significantly affect the optical properties of the hydrogel matrix.^16^ For comparison the corresponding value of human corneas lie between 1.373 to 1.380 ^22^ while the commercial AlphaCor-manufactured from a single polymer of pHEMA exhibits refractive index of 1.43.^23^ All of these values fall within the range previously reported for related pHEMA-based materials developed by our group, including pHEMA@AGMNA (AGMNA = {[Ag_6_(µ_3_-HMNA)_4_(µ_3_-MNA)_2_]^2−^·[(Et_3_NH)^+^]_2_·(DMSO)_2_·(H_2_O)}, H_2_MNA = 2-thionicotinic acid), which exhibited a refractive index of 1.436 ^16^pHEMA@AGGLY-2 (AGGLY = [Ag_3_(Gly)_2_NO_3_]_*n*_, GlyH = glycine), with a refractive index of 1.4335; pHEMA@AGU-2 (AGU = [Ag(U)NO_3_]_*n*_, U = urea), with a refractive index of 1.4345; and pHEMA@AGSAL-2 ([Ag(salH)]_2_, AGSAL; salH_2_ = salicylic acid), with a refractive index of 1.437. These findings further demonstrate that incorporation of silver-based antimicrobial agents into the pHEMA matrix has a negligible effect on its optical properties.^23^

### X-ray fluorescence spectroscopy

**Fig. 1:**
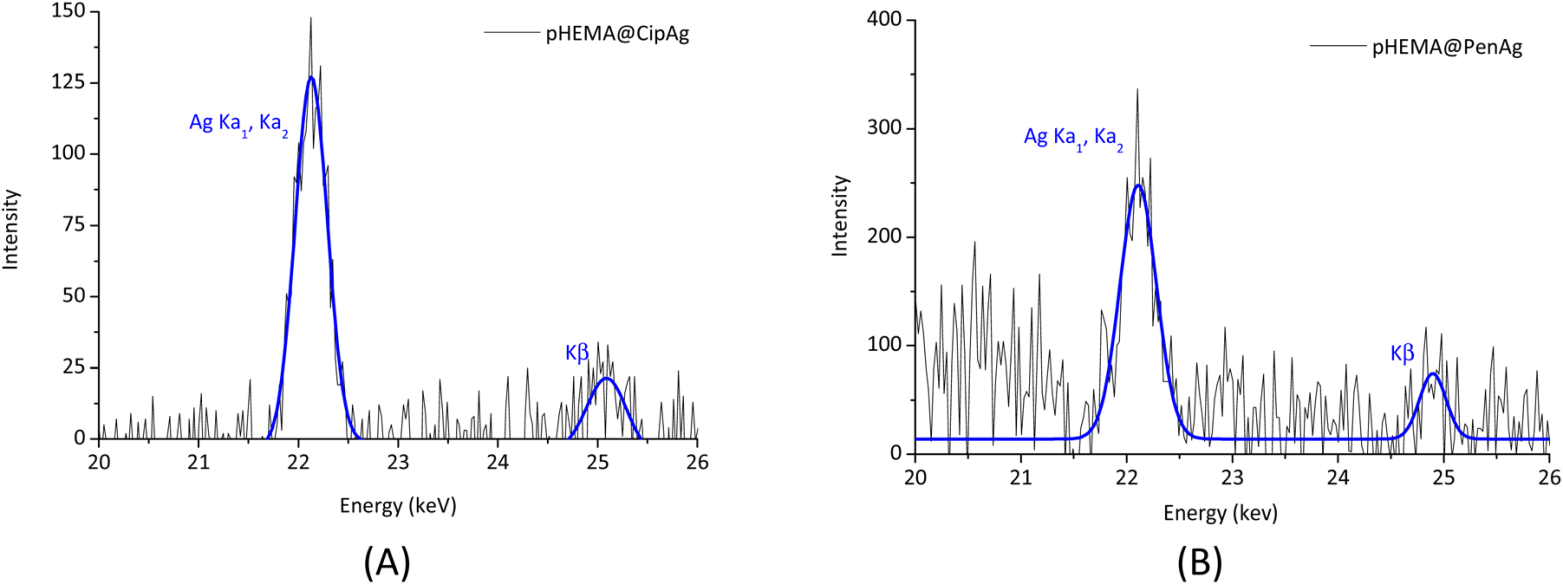
XRF spectra of pHEMA@CipAg (A) and pHEMA@PenAg (B).

The XRF spectra of dry pHEMA@CipAg and pHEMA@PenAg discs were recorded to confirm the presence of Ag within the materials (Fig. 1). The silver content in pHEMA@CipAg and pHEMA@PenAg was determined to be 0.0254 and 0.0499% w/w, respectively, while the theoretical Ag content in the discs was calculated to be 0.0210 and 0.0418% w/w respectively. The agreement between the experimental and theoretical Ag values confirms the successful encapsulation of CipAg and PenAg into g the pHEMA hydrogel network.

### Solid state UV-vis spectra

**Fig. 2:**
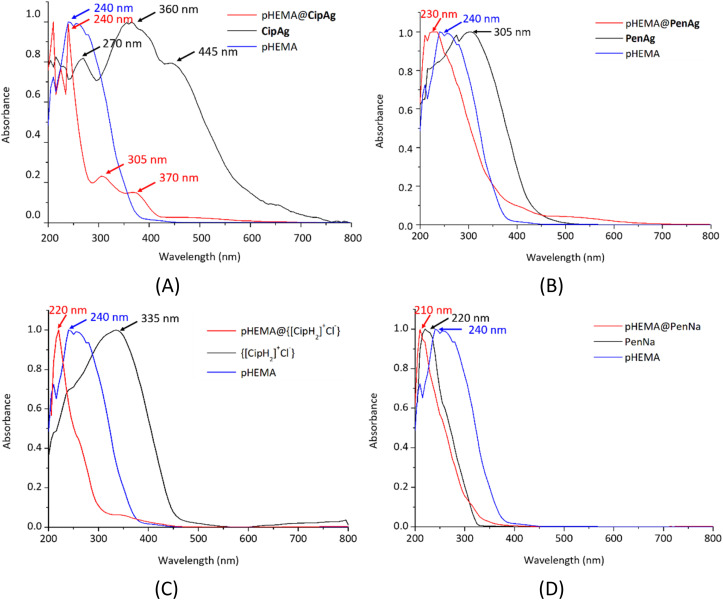
Solid-state UV-vis spectra of pHEMA compared with pHEMA@CipAg and CipAg (A), pHEMA@PenAg and PenAg (B), pHEMA@{[CipH_2_]^+^Cl^−^} and {[CipH_2_]^+^Cl^−^} (C), as well as pHEMA@penNa and penNa (D).

The successful dispersion of the metallonantibiotics (CipAg and PenAg) and their corresponding precursors {[CipH_2_]^+^Cl^−^} and PenNa) within pHEMA matrices (pHEMA@CipAg, pHEMA@PenAg, pHEMA@{[CipH_2_]^+^Cl^−^}, and pHEMA@PenNa) was confirmed by solid-state UV-vis spectroscopy (Fig. 2). Based on the solid-state UV-vis spectra of the prepared materials, the incorporation of the metalloantibiotics into the pHEMA matrix resulted in significant spectral changes compared to pure pHEMA, confirming the successful loading of the complexes into the polymeric network. Pure pHEMA exhibited a characteristic absorption band at approximately 240 nm, which is attributed to π → π* transitions of the polymer backbone. Free CipAg display absorption bands at 445, 360, and 270 nm. In contrast, the pHEMA@CipAg materials displayed broad absorption bands centered at 370, 305 and 240 nm, arising from ligand-centered and charge–transfer transitions associated with the CipAg. The absorption bands in the solid state Uv-vis spectrum of PenAg is observed at 305 nm. The pHEMA@PenAg materials showed a distinct absorption band at 230 nm, which can be attributed to electronic transitions of the corresponding PenAg complex. The solid-state UV-vis spectra of {[CipH_2_]^+^Cl^−^} and PenNa show absorption bands at 335 and 220 nm respectively. The pHEMA materials containing the precursor antibiotics also exhibited characteristic absorption bands corresponding to the incorporated drugs. Specifically, pHEMA@{[CipH_2_]^+^Cl^−^}, showed a characteristic absorption band at 220 nm, while pHEMA@PenNa displayed absorption bands at 210 nm, confirming the successful incorporation of the parent antibiotics into the polymeric matrix as well. The observed hypsochromic shifts and broadening of the absorption bands after incorporation into the pHEMA matrix indicate strong interactions between the polymeric network and the embedded metalloantibiotics and their free antibiotics, while also suggesting the homogeneous distribution of the complexes within the hydrogel structure.

### Differential scanning calorimetry analysis of pHEMA@CipAg, pHEMA@PenAg, pHEMA@{[CipH_2_]^+^Cl^−^}, and pHEMA@PenNa

To examine whether the newly developed systems constitute composite materials rather than simple physical mixtures of the starting components, DSC thermograms were recorded and comparatively analyzed (Fig. S1). In the DSC thermogram of pHEMA, the characteristic phase transition is observed at 269 °C. Upon incorporation of CipAg, PenAg, {[CipH_2_]^+^Cl^−^}, and PenNa into the polymeric matrix, this transition occurs to lower temperatures, namely 245, 245, 226, and 233 °C for pHEMA@CipAg, pHEMA@PenAg, pHEMA@{[CipH_2_]^+^Cl^−^}, and pHEMA@PenNa, respectively. These pronounced thermal shifts strongly indicate intermolecular interactions between the incorporated agents and the pHEMA matrix, confirming the successful formation of integrated composite materials rather than simple physical mixtures.

### Biological tests

Since the reported metalloantibiotics, together with their corresponding parent antibiotics, exhibit significant antimicrobial activity against *P. aeruginosa*, *S. epidermidis*, and *S. aureus* (Table 1), they were incorporated into the pHEMA matrix for the fabrication of therapeutic contact lenses.^14,17^ Subsequently, contact lens discs with diameter 10 mm, containing the agents at a concentration of 1 mM were used in all biological experiments.

**Table 1:** Bacterial viability (%) and Human Corneal Epithelial Cell (HCECs) viability (%), together with the inhibition zones (IZ), of pHEMA@CipAg, pHEMA@{[CipH_2_]^+^Cl^−^}, pHEMA@PenAg, pHEMA@PenNa, and their corresponding precursors against *P. aeruginosa*, *S. epidermidis*, and *S. aureus*^a^

	*P. aeruginosa*	*S. epidermidis*	*S. aureus*	Ref.
**Bacteria viability (%)**				
pHEMA@CipAg	1.3 ± 0.7	0.4 ± 0.0	1.4 ± 0.2	*
pHEMA@{[CipH_2_]^+^Cl^−^}	0.2 ± 0.2	0.5 ± 0.3	0.8 ± 0.4	*
pHEMA@PenAg	77.9 ± 6.5	78.3 ± 5.1	9.0 ± 4.8	*
pHEMA@PenNa	91.9 ± 7.1	92.8 ± 15.2	99.8 ± 0.5	*

**MIC (µM)**				
CipAg	0.61 ± 0.14	0.46 ± 0.08	0.54 ± 0.07	14
{[CipH_2_]^+^Cl^−^}	1.17 ± 0.22	1.08 ± 0.12	1.45 ± 0.12	14
PenAg	23.00 ± 2.29	2.41 ± 0.88	0.08 ± 0.02	17
PenNa	>2000	3.76 ± 1.14	0.14 ± 0.02	17

**IZ (mm)**				
pHEMA@CipAg	41.0 ± 4.0	32.0 ± 4.0	30.0 ± 3.0	*
pHEMA@{[CipH_2_]^+^Cl^−^}	36.7 ± 4.0	30.0 ± 2.3	28.7 ± 4.0	*
pHEMA@PenAg	11.0 ± 0.8	12.0 ± 1.4	18.7 ± 2.8	*
pHEMA@PenNa	10.0 ± 0.0	10.4 ± 0.8	10.7 ± 1.3	*

**Elimination of preformed biofilm (%)**				
pHEMA@CipAg	66.7 ± 11.3	—	77.6 ± 5.8	*
pHEMA@{[CipH_2_]^+^Cl^−^}	40.6 ± 13.2	—	69.7 ± 10.2	*
pHEMA@PenAg	70.0 ± 9.7	—	21.8 ± 11.1	*
pHEMA@PenNa	30.0 ± 6.2	—	22.2 ± 11.1	*

**HCEC cell viability (%)**				
pHEMA@CipAg	71.6 ± 3.2			*
pHEMA@{[CipH_2_]^+^Cl^−^}	79.5 ± 1.9			*
pHEMA@PenAg	81.8 ± 8.3			*
pHEMA@PenNa	98.3 ± 2.3			*

a *in this study.

### Antimicrobial activity of functionalized pHEMA-based materials in liquid broth medium

**Fig. 3:**
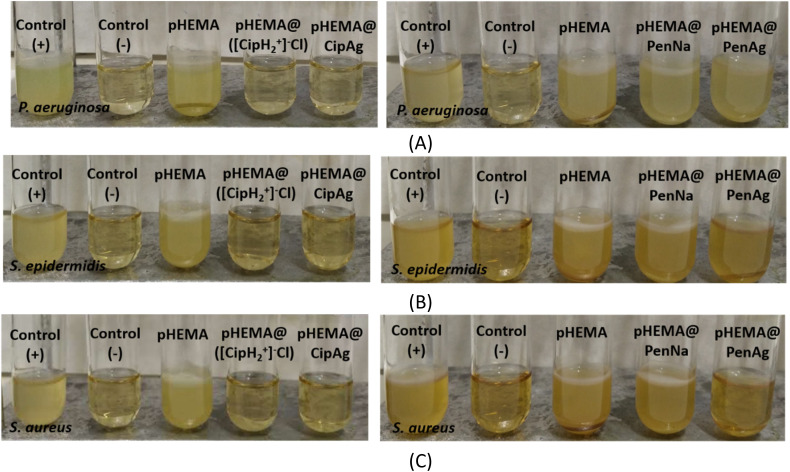
Bacterial strains of *P. aeruginosa*, *S. epidermidis*, and *S. aureus* viability upon there incubation a for 20 h with the disks of pHEMA, pHEMA@CipAg, pHEMA@{[CipH_2_]^+^Cl^−^}, pHEMA@PenAg, and pHEMA@PenNa

Bacterial strains of *P. aeruginosa*, *S. epidermidis*, and *S. aureus* were incubated for 20 h with the disks of pHEMA, pHEMA@CipAg, pHEMA@{[CipH_2_]^+^Cl^−^}, pHEMA@PenAg, and pHEMA@PenNa (Fig. 3).

In the case of the pHEMA@CipAg and pHEMA@{[CipH_2_]^+^Cl^−^} disks, the calculated bacterial viability percentages were remarkably low, ranging from 0.2–1.3% against *P. aeruginosa*, 0.4–0.5% against *S. epidermidis*, and 0.8–1.4% against *S. aureus* (Table 1). In contrast, the disks of pHEMA@PenAg (9.0–78.3%) and pHEMA@PenNa (91.9–99.8%) exhibited substantially higher bacterial viability values against the aforementioned bacterial strains. Similarly, the previously reported biomaterials pHEMA@AGGLY-2, pHEMA@AGU-2, and pHEMA@AGSAL-2 also exhibited low bacterial viability, with values ranging from 0.8–1.2%, 2.1–5.2%, and 2.4–3.3%, respectively, against *P. aeruginosa*, *S. epidermidis*, and *S. aureus*.^19^

**Fig. 4:**
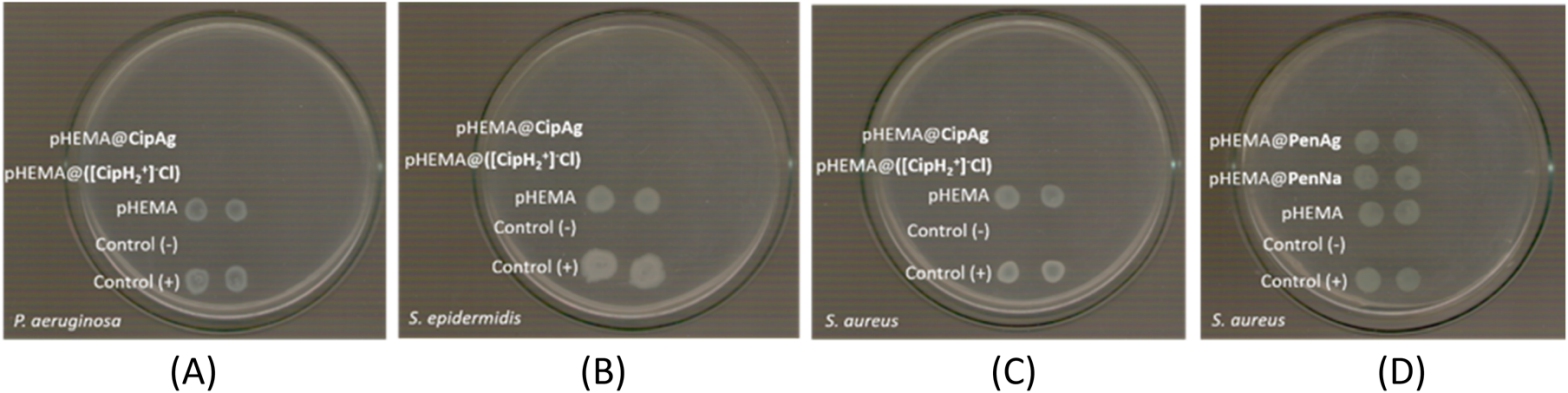
Bacterial colonies of *P. aeruginosa* (A), *S. epidermidis* (B), and *S. aureus* (C, D) grown on agar plates using supernatants obtained from solutions initially treated with discs of pHEMA, pHEMA@CipAg, pHEMA@{[CipH_2_]^+^Cl^−^} (A–C), and pHEMA@PenAg (D).

Furthermore, the bacterial suspensions of *P. aeruginosa*, *S. epidermidis,* and *S. aureus* obtained from the broth cultures containing the pHEMA@CipAg and pHEMA@{[CipH_2_]^+^Cl^−^} discs, as well as the *S. aureus* suspension from the broth culture containing the pHEMA@PenAg disc, were transferred onto Petri dishes and incubated for an additional 20 h (Fig. 4). In the case of pHEMA@CipAg and pHEMA@{[CipH_2_]^+^Cl^−^}, no bacterial colony formation was observed. In contrast, visible bacterial colonies were detected for the pHEMA@PenAg disks, indicating that this material does not induce permanent bacterial inhibition and may therefore allow preservation of the natural microbial flora. In contrast, the materials pHEMA@AGGLY-2, pHEMA@AGU-2, and pHEMA@AGSAL-2 inhibit microbial growth only while they are present in the microbial cultures.^19^

### Inhibition zones (IZ) – solid agar disc diffusion

**Fig. 5:**
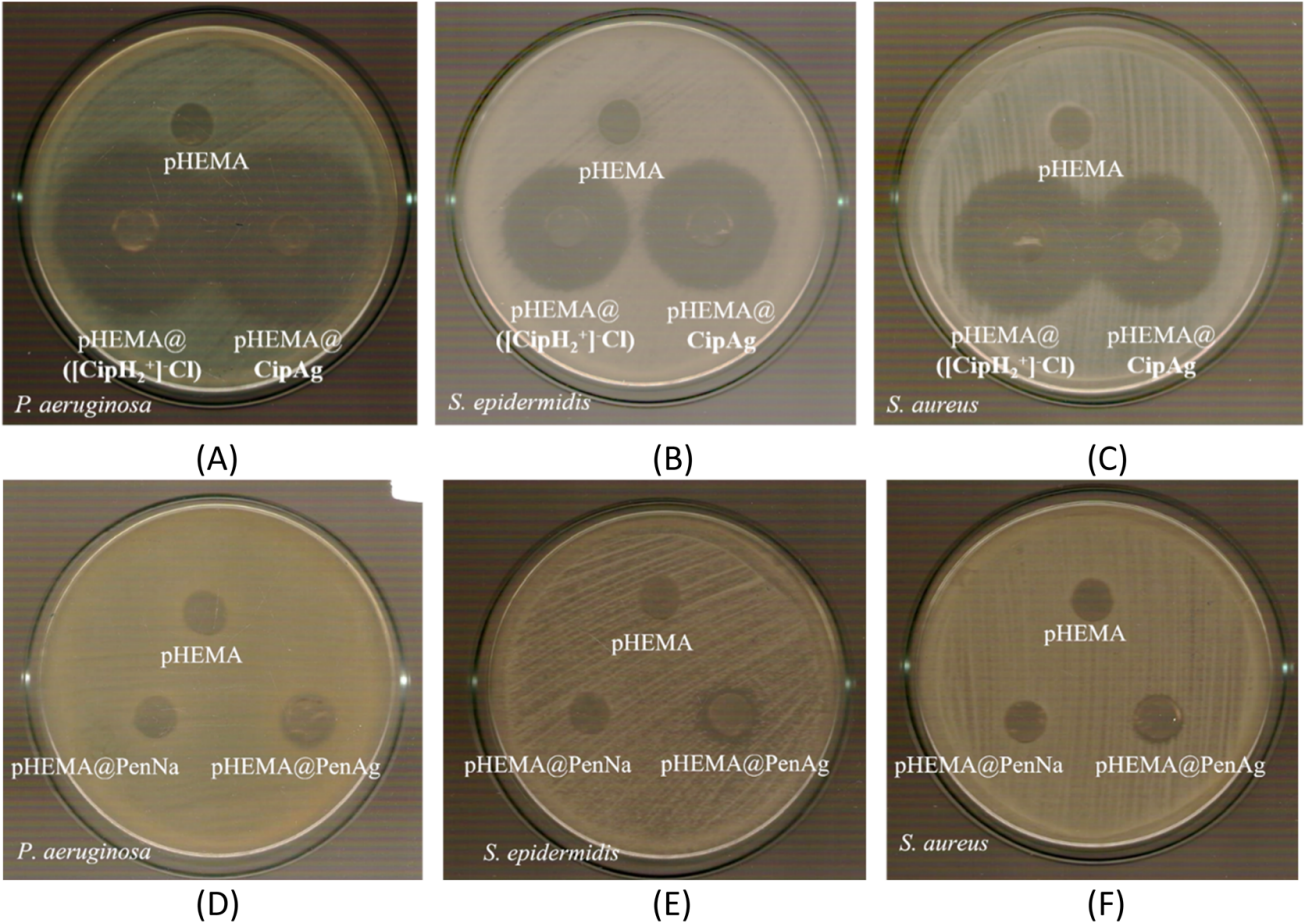
IZs which are developed in agar plates of *P. aeruginosa*, *S. epidermidis* and *S. aureus* by (A–C) pHEMA@CipAg, pHEMA@([CipH_2_^+^]^−^Cl), (D–F) pHEMA@PenAg and pHEMA@PenNa.

The antibacterial activity of the materials was also evaluated using the agar disc diffusion method against *P. aeruginosa*, *S. epidermidis* and *S. aureus* following 20 h of incubation (Table 1 and Fig. 5). Both metalloantibiotic-based materials, pHEMA@CipAg and pHEMA@PenAg, exhibited substantially larger IZ's than the corresponding materials containing the parent antibiotics alone. In particular, pHEMA@CipAg displayed IZ values ranging from 30.0 to 41.0 mm, compared to 28.7 to 36.7 mm for pHEMA@{[CipH_2_]^+^Cl^−^}. In contrast, the IZ's developed around the discs treated with the pHEMA@PenAg solution ranged from 11.0 to 18.7 mm, whereas the corresponding values for pHEMA@PenNa were limited to 10.0–10.7 mm. It is noteworthy that pHEMA@PenAg exhibited antibacterial activity against the Gram-negative strain *P. aeruginosa*, despite the complete inactivity of PenNa and the very limited activity of pHEMA@PenNa (10.0–10.7 mm) against Gram-negative microorganisms. The inhibition zones produced by pHEMA@AGGLY-2, pHEMA@AGU-2, and pHEMA@AGSAL-2 discs ranged from 12.2 to 15.2 mm against *P. aeruginosa*, *E. coli*, *S. epidermidis*, and *S. aureus*.^19^

According to the classification criteria based on inhibition zone (IZ) values, microbial strains exhibiting IZ ≥ 17 mm are considered susceptible, whereas strains showing IZ ≤ 12 mm are regarded as resistant against an agent.^14^ Those with IZ values between 13 and 16 mm (13 ≤ IZ ≤ 16 mm) are classified as intermediate.^14^ According to this classification, the microbial strains *P. aeruginosa*, *S. epidermidis*, and *S. aureus* are characterized as highly susceptible to pHEMA@CipAg and pHEMA@{[CipH_2_]^+^Cl^−^} agents, as all recorded IZ values were ≥ 17 mm. This highlights the remarkable antimicrobial efficacy of these metalloantibiotics (Table 1).

### Effect on biofilm formation

**Fig. 6:**
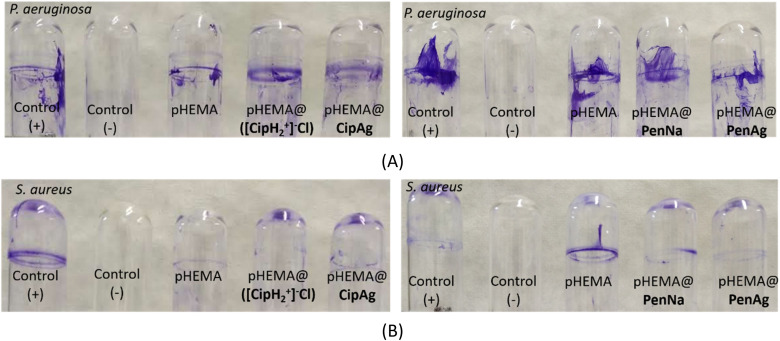
Biofilms of *P. aeruginosa* (A) and *S. aureus* (B) elimination caused by pHEMA@CipAg, pHEMA@([CipH_2_^+^]^−^Cl), pHEMA@PenAg and pHEMA@PenNa.

Bacteria can adhere to contact lens materials and survive within storage cases, mainly due to their ability to form resistant biofilms on both the lenses and the cases.^19,24^ Ophthalmic biofilm-associated infections are particularly difficult to treat, while surgical removal of the infected tissue carries a substantial risk of corneal blindness. For this purpose, the biofilm-forming strains *P. aeruginosa* and *S. aureus* were cultured in test tubes for 24 h at 37 °C. Subsequently, the materials pHEMA@CipAg, pHEMA@{[CipH_2_]^+^Cl^−^}, pHEMA@PenAg, and pHEMA@PenNa were added and incubated for an additional 20 h, while the percentage of preformed biofilm removal was evaluated using the crystal violet assay (Fig. 6).

The disks of pHEMA@CipAg, pHEMA@{[CipH_2_]^+^Cl^−^}, pHEMA@PenAg, and pHEMA@PenNa eliminate the preformed biofilm of *P. aeruginosa* by 66.7, 40.6, 70.0, and 30.0%, respectively, indicating the high potency of the metalloantibiotic-based materials. However, in the case of *S. aureus*, the biofilm removal percentages were comparable to those observed for the corresponding antibiotic-based materials, namely 77.6% for pHEMA@CipAg, 69.7% for pHEMA@{[CipH_2_]^+^Cl^−^}, 21.8% for pHEMA@PenAg, and 22.2% for pHEMA@PenNa. The percentages of preformed biofilm elimination by pHEMA@AGGLY-2, pHEMA@AGU-2, and pHEMA@AGSAL-2 discs ranged from 27.5 to 40.3% against *P. aeruginosa* and from 23.2 to 42.0% against *S. aureus*.^19^

### *In vitro* toxicity and genotoxicity against human corneal epithelial cells (HCEC)

The ocular toxicity of the new contact Lense disks was evaluated against HCEC cells upon their incubation for a period of 24 h.^14–21,25^ The calculated viability was 71.6 ± 3.2 for pHEMA@CipAg, 79.5 ± 1.9 for pHEMA@{[CipH_2_]^+^Cl^−^}, 81.8 ± 8.3 for pHEMA@PenAg and 98.3 ± 2.3% for pHEMA@PenNa. According to ISO 10993-5 for the biological evaluation of medical devices, materials exhibiting cell viability values above 70% are considered non-cytotoxic.^26^ Therefore, the above-mentioned materials are classified as low-toxic or non-cytotoxic ones. The corresponding cell viability values after 24 h of incubation were 84.2 ± 1.9%, 98.8 ± 2.7%, and 95.4 ± 3.0% for pHEMA@AGGLY-2, pHEMA@AGU-2, and pHEMA@AGSAL-2, respectively.^19^

**Fig. 7:**
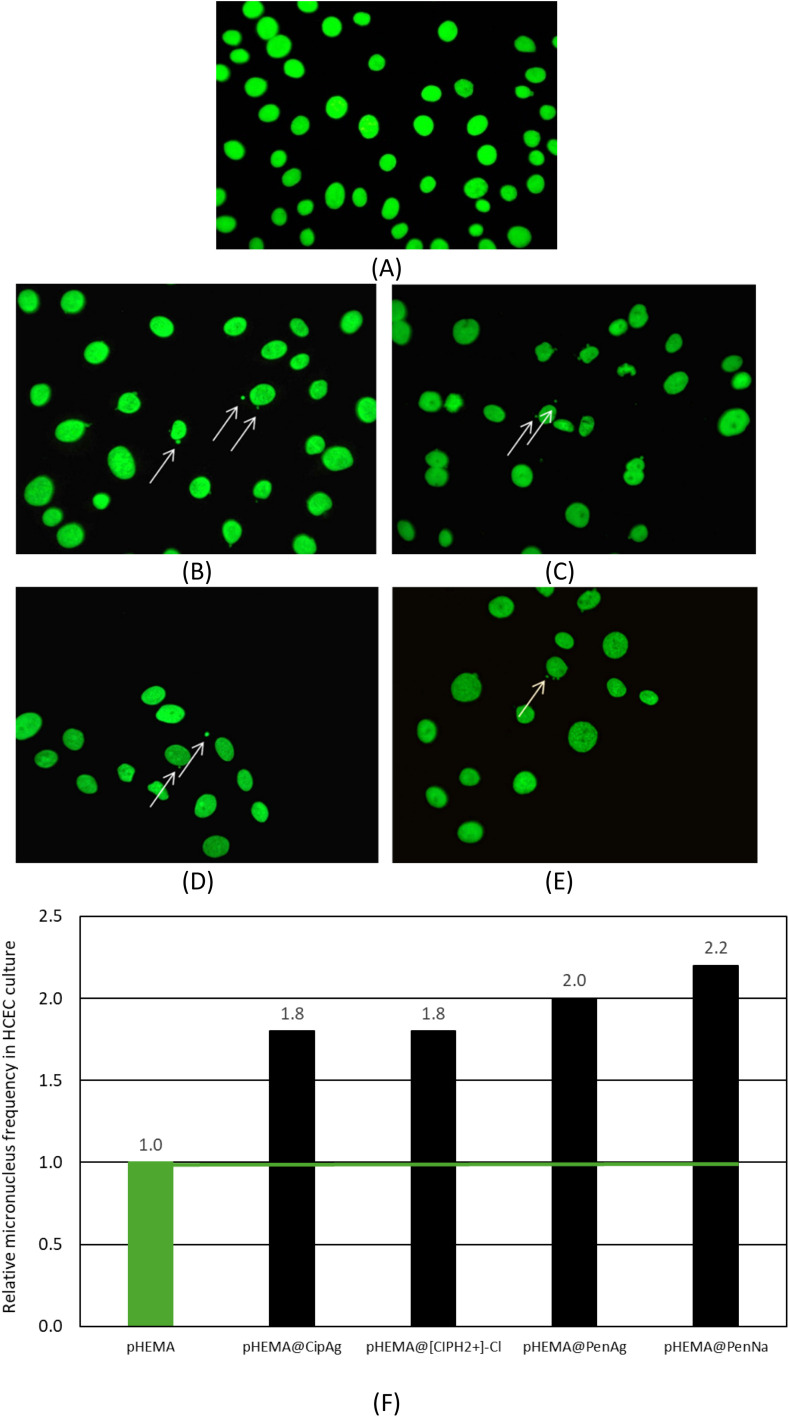
Micronucleus formed in upon their treatment with pHEMA (A), pHEMA@CipAg (B), pHEMA@[CipH_2_^+^]^−^Cl (C) pHEMA@PenAg (D) and pHEMA@PenNa (E), for a period of 24 h; the arrow indicates the micronucleus in HCECs. Relative micronucleus frequency in HCEC cells culture (F).

In order to quantify the chromosomal damage, the MN assay was used to screen chemicals for genetic toxicity.^27–29^ The presence of MN, which result from chromosomal breakage and/or chromosome loss, may serve as a biomarker of potential mutagenic, genotoxic, or teratogenic effects. The HCEC cells were incubated in the presences of disks for 24 h and the MN frequency was calculated, using fluorescence microscope (Fig. 7). In the case of materials, the percentage of MN are 4.5 ± 0.9 for pHEMA@CipAg, 4.5 ± 1.4 for pHEMA@{[CipH_2_]^+^Cl^−^}, 5.1 ± 1.2 for pHEMA@PenAg, 5.5 ± 0.3 for pHEMA@PenNa, in contrast to treated cells with pHEMA 2.5 ± 0.5%. Low *in vitro* genotoxic effects were previously observed for pHEMA@AGGLY-2, pHEMA@AGU-2, and pHEMA@AGSAL-2, with MN frequencies ranging from 2.1 to 2.4%, compared with 1.3% for HCEC cells treated with pHEMA.^19^

### *In vivo* toxicity using *Artemia salina* assay

*Artemia salina* is widely used as an early indicator of biological safety due to its short life cycle and high sensitivity to ionic and chemical stressors.^30,31^ Furthermore, the U.S. Environmental Protection Agency (EPA) recognizes *A. salina* as a suitable model organism for *in vivo* toxicological screening of bioactive compounds and pharmaceuticals.^32^

The *in vivo* toxicity of the developed materials was evaluated using the *Artemia salina* lethality assay. Following incubation of brine shrimp larvae with pHEMA, pHEMA@CipAg, pHEMA@{[CipH_2_]^+^Cl^−^}, pHEMA@PenAg, and pHEMA@PenNa for 24 h, no mortality was observed in any of the tested groups. These findings indicate that the materials exhibit negligible acute toxicity under the experimental conditions employed and support their favorable biocompatibility profile. Comparable findings were also observed for pHEMA@AGGLY-2, pHEMA@AGU-2, and pHEMA@AGSAL-2.^19^

### *In vivo* genotoxicity study with the *Allium cepa* assay

**Fig. 8:**
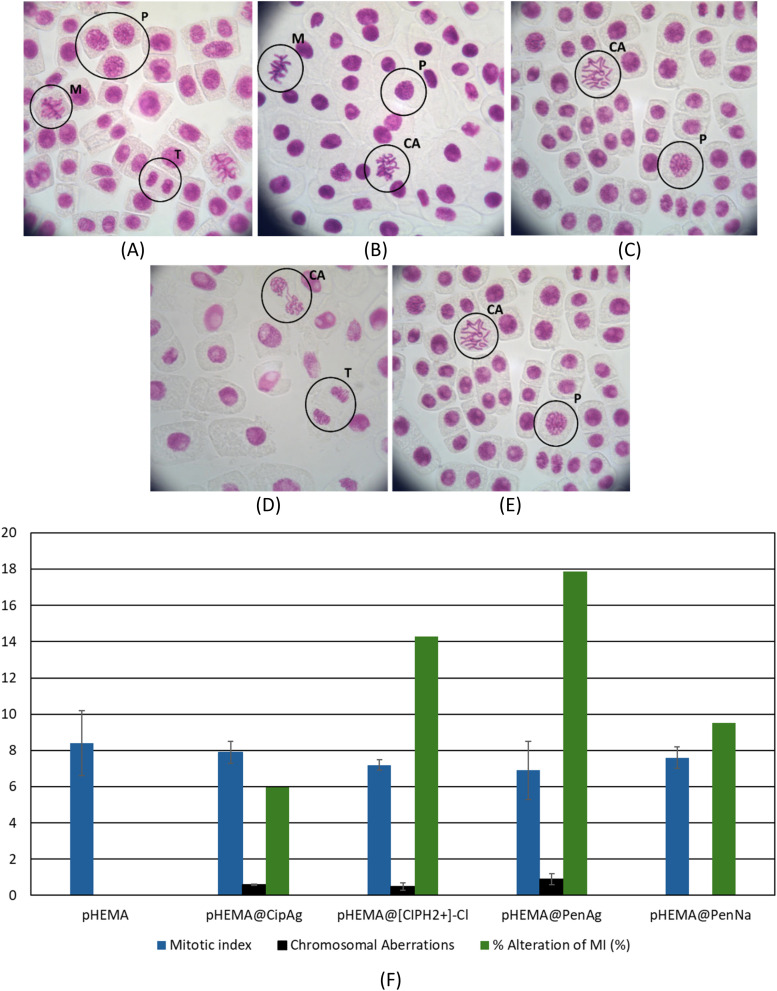
*Allium cepa* meristematic cells exposed to materials. *Allium cepa* meristematic cells exposed with pHEMA (A), pHEMA@CipAg (B), pHEMA@[CipH_2_^+^]^−^Cl (C), pHEMA@PenAg (D) and pHEMA@PenNa (E) (P = prophase, A = anaphase, M = metaphase, T = telophase, CA = chromosomal aberrations). Diagram (F) of mitotic index% (MI), chromosomal aberrations% (CA) and% mitotic index alteration (MIA (%)) observed when *Allium cepa* was incubated with materials.

*Allium cepa* is an *in vivo* model, suitable for toxicity and genotoxicity evaluation of drugs.^27^ It is a bioindicator model due to its large genome size and low number of chromosomes.^27,33^ The Environmental Protection Agency (EPA) and the World Health Organization acknowledge that the data from this bioassay are effective and reliable in the determination of genotoxicity.^34^ Bioindicator parameters are investigated as mitotic index (MI), micronuclei (MN), and chromosomal aberrations (CA). The MI (%) and DNA damages such as CA (%) are calculated when roots of *Allium cepa* are treated with materials for 48 h (Fig. 8). Moreover, the % alteration of the MI (% MIA) has been defined, and it is calculated as the MI of the treated *Allium cepa* cells toward the MI of the untreated ones (% MIA = (100 × (MI_treated_ − MI_untreated_))/(MI_untreated_).^35^

The MI in the case of treated roots of *A. cepa* with pHEMA is 8.4 ± 1.8 and it slight decreased 7.9 ± 0.6 (pHEMA@CipAg), 7.2 ± 0.3 (pHEMA@[CipH2+]−Cl), 6.9 ± 1.6 (pHEMA@PenAg) and 7.6 ± 0.6 (pHEMA@PenNa). Moreover, the percentage of CA is slight increase in the case of pHEMA@CipAg (0.6 ± 0.1), pHEMA@{[CipH_2_]^+^Cl^−^} (0.5 ± 0.2) and pHEMA@PenAg (0.9 ± 0.3), in contrast to no detectable CA in the case of pHEMA and pHEMA@PenNa. The calculated MIA (%) is 6.0, 14.3, 17.9 and 9.5% for pHEMA@CipAg, pHEMA@{[CipH_2_]^+^Cl^−^}, pHEMA@PenAg and pHEMA@PenNa, respectively. According to ISO 10993-5 these new materials are considered as nontoxic. The MI and CA values observed for pHEMA@AGGLY-2, pHEMA@AGU-2, and pHEMA@AGSAL-2 were comparable to those of the pHEMA-treated group, indicating the absence of *in vivo* genotoxicity.^19^

## Conclusion

The delivery of medications to the eye can be achieved by topical application, intravitreal injection and rarely by the systemic route.^36^ Each of these drug delivery methods possess low bioavailability and poor drug delivery efficiency.^36^ Mostly, the residence time of the drug in the tear film is only a few minutes, and therefore the bioavailability of the drug is about 1–5%.^36^ Contact lenses have been used for ocular drug delivery due to high bioavailability and continuous drug release to the target tissue, without causing visual disturbance.^36^ This increases the retention time of the drug at the corneal level (about 30 min) as opposed to around 2 min in the case of eye drops.^36^ The therapeutic contact lenses are used to deliver ophthalmic drugs and the therapeutic efficacy can be improved compared to eye drops.^36^ Several strategies are used as therapeutic contact lenses including nanoparticles, micelles, liposomes-laden contact lenses, biomimetic imprinted contact lenses, vitamin E barriers in contact lenses.^36^ Despite all the *in vivo* studies, commercial therapeutic contact lenses are rarely available today.^36^

Compared with previously reported antimicrobial and therapeutic contact lenses, the metalloantibiotic-functionalized material pHEMA@CipAg exhibited remarkably enhanced antibacterial and antibiofilm activity. In particular, pHEMA@CipAg demonstrated extremely low bacterial viability values against *P. aeruginosa*, *S. epidermidis*, and *S. aureus* (1.3, 0.4, and 1.4%, respectively), indicating near-complete inhibition of bacterial. Similar ciprofloxacin-loaded hydrogel or silicone hydrogel contact lenses reported in the literature generally achieve partial bacterial suppression, frequently leaving significant residual bacterial survival, especially against biofilm-forming *P. aeruginosa* strains.^37–39^

Furthermore, pHEMA@CipAg exhibited large inhibition zones (IZs) ranging from 30.0 to 41.0 mm against all tested strains. These IZ values are considerably higher than those reported for most conventional ciprofloxacin-loaded hydrogel lenses, silver-coated lenses, or nanoparticle-modified therapeutic lenses, where inhibition zones are commonly reported within the range of approximately 10–25 mm depending on the microorganism and release profile.^40^ The particularly large IZ observed for *P. aeruginosa* (41.0 mm) indicates very efficient antimicrobial diffusion and sustained activity of the metalloantibiotic-functionalized material.

The antibiofilm activity of pHEMA@CipAg was also highly significant, eliminating 66.7% of preformed *P. aeruginosa* biofilm and 77.6% of *S. aureus* biofilm. This performance compares favorably with many reported antibiofilm ophthalmic materials and antimicrobial coatings, which often mainly inhibit initial adhesion rather than effectively removing mature preformed biofilms.^41^ Biofilm inhibition remains one of the greatest challenges in ocular infections because bacteria embedded within biofilms exhibit dramatically increased resistance to antibiotics and other antimicrobial agents.^42,43^

Importantly, pHEMA@CipAg combines three highly desirable therapeutic properties simultaneously: (i) near-complete suppression of bacterial viability, (ii) exceptionally large inhibition zones, and (iii) strong elimination of established biofilms. Such a multifunctional antimicrobial profile is rarely achieved in currently available therapeutic contact lens systems, which usually emphasize either prolonged antibiotic release or prevention of microbial adhesion, but not efficient inhibition of mature microbial biofilms.

## Experimental

### Materials and instruments

All solvents used were of reagent grade and were used with no further purification. Silver nitrate was purchased from Degussa (Berlin, Germany). Melting points were measured in open tubes with Stuart Scientific apparatus and are uncorrected. Mid infrared spectra (4000–400 cm^−1^) were obtained on Cary 670 FTIR spectrometer (Agilent Technologies). 2-Hydroxyethyl-methacrylate (pHEMA), ethylene-glycole-dimethacrylate (EGDMA, Merck), diphenyl (2,4,6-trimethylbenzoyl) phosphine oxide (TPO 97%, Sigma-Aldrich) as well as sodium chloride (NaCl, Merck) and hydrochloric acid (HCl 37%, Merck) were used. XRF measurement was also carried out with a Rigaku NEX QC EDXRF analyzer (Austin, TX, USA) Tryptone tryptophan medium, beef extract powder, peptone bacteriological, soy peptone were purchased from Biolife. Agar and yeast extract were purchased from Fluka Analytical. Sodium cloride, d(+)-glucose, di-potassium hydrogen phosphate trihydrate were purchased from Merck. Dulbecco's modified Eagle's medium (DMEM), fetal bovine serum, and phosphate buffer saline (PBS) were purchased from Sigma-Aldrich. Trypsin-EDTA and l-glutamine were purchased from Biowest. Penicillin-streptomycin was purchased from Gibco, Glasgow, UK. Sulforhodamine B was purchased from Alfa Aesar. Dimethyl sulfoxide was purchased from Riedel-de Haën. For the toxicity experiments, brine shrimp eggs (*Artemia salina*) were purchased from Ocean Nutrition. Sea salt was purchased from Tropic Marin. Fresh onion bulbs (*Allium cepa*), with diameter of ∼1.0–1.5 cm.

### Synthesis and crystallization of CipAg and PenAg

CipAg and PenAg were synthesized as described in a previous work.^14,17^

### Synthesis of pHEMA@CipAg, pHEMA@{[CipH_2_]^+^Cl^−^}, pHEMA@PenAg and pHEMA@PenNa

Hydrogels of pHEMA with incorporated CipAg, {[CipH_2_]^+^Cl^−^}, PenAg and PenNa were obtained as follows: 2.7 mL of HEMA was mixed with 2 mL of double distilled water (ddw), which contains CipAg, {[CipH_2_]^+^Cl^−^}, PenAg and PenNa (1 mM) and 10 µL of EGDMA. The solution was then degassed by bubbling with nitrogen for 15 minutes. TPO initiator (6 mg) was added to the solution and mixed for 5 min at 800 rpm. The solution was poured into the mold and was then placing under a UV mercury lamp (*λ*_max_ = 280 nm), 15 watt, where photopolymerization occurred, for 40 minutes. Unreacted monomers were removed, by immersed the gel in boiling water for 15 min. Discs with 10 mm diameter were cut, and they washed by immersion in water, NaCl 0.9%, HCl 0.1 M, and again in water. The discs were then dried at 40 °C until no weight change would occur.

### Differential scanning calorimetry (DTG/DSC)

The measurements were performed in a DTG/TG NETZSCH STA 449C. For the measurements, 3 mg of materials were placed inside a platinum capsule with-alumina to be the reference sample. The temperature increasing rate was 10 °C min in the range of 25–400 °C and the measurements were performed in air atmosphere. The samples measured were in fine-grained powder form.

### Biological tests

#### Bacterial strains

The bacterial strains of *P. aeruginosa*, *S. aureus* (ATCC® 25923™), and *S. epidermidis* (ATCC® 14990™), were adopted in the experiments. The biological experiments were performed in triplicates. Effects on the growth of microbial strains, viability of microbes upon incubated with pHEMA, pHEMA@CipAg, pHEMA@{[CipH_2_]^+^Cl^−^}, pHEMA@PenAg and pHEMA@PenNa, IZ and removal of preformed biofilm were performed as previously reported.^14,17,19^*In vitro* toxicity using HCEC cells, *in vitro* genotoxicity using HCEC cells, *in vivo* toxicity with Brine shrimp assay and *in vivo* genotoxicity with *Allium cepa* were performed as previously reported.^14,17,19^

## Author contributions

Conceptualization, S. K. H.; supervision, S. K. H.; investigation; methodology, C. N. B. and S. K. H.; validation, S. K. H.; writing – original draft, C. N. B. and S. K. H.; writing – review and editing, C. N. B. and S. K. H. All authors have read and agreed to the published version of the manuscript.

## Conflicts of interest

There are no conflicts to declare by the authors.

## Supplementary Material

RA-OLF-D6RA04865J-s001

## Data Availability

The data supporting the findings of this study are available within the article and its supplementary information (SI). Additional raw data (spectroscopic files) are available from the corresponding author upon reasonable request. Supplementary information is available. See DOI: https://doi.org/10.1039/d6ra04865j.
