# Supplementary material for: Metalloantibiotic functionalized pHEMA for therapeutic contact lenses
Source: RSC Adv. 2026 Aug 7;16(43):52355–67. doi: 10.1039/d6ra04865j (PMC13448763; doi:10.1039/d6ra04865j)
Supplement: Supplementary file 1 [file RA-016-D6RA04865J-s001.pdf]

Supplementary Information (SI) for RSC Advances.
This journal is © The Royal Society of Chemistry 2026

Electronic Supplementary Information

Metalloantibiotic Functionalized pHEMA for Therapeutic Contact Lenses

Sotiris K. Hadjikakou[1,*] and Christina N. Banti[1,*],

[1] Biological Inorganic Chemistry Laboratory, Department of Chemistry, University of Ioannina, 45110 Ioannina, Greece

*All correspondence should be addressed to:

Dr. S.K. Hadjikakou (Professor); e-mail: shadjika@uoi.gr; tel. x30-26510-08374, x30-26510-08362

Dr. C.N. Banti; email: cbanti@uoi.gr

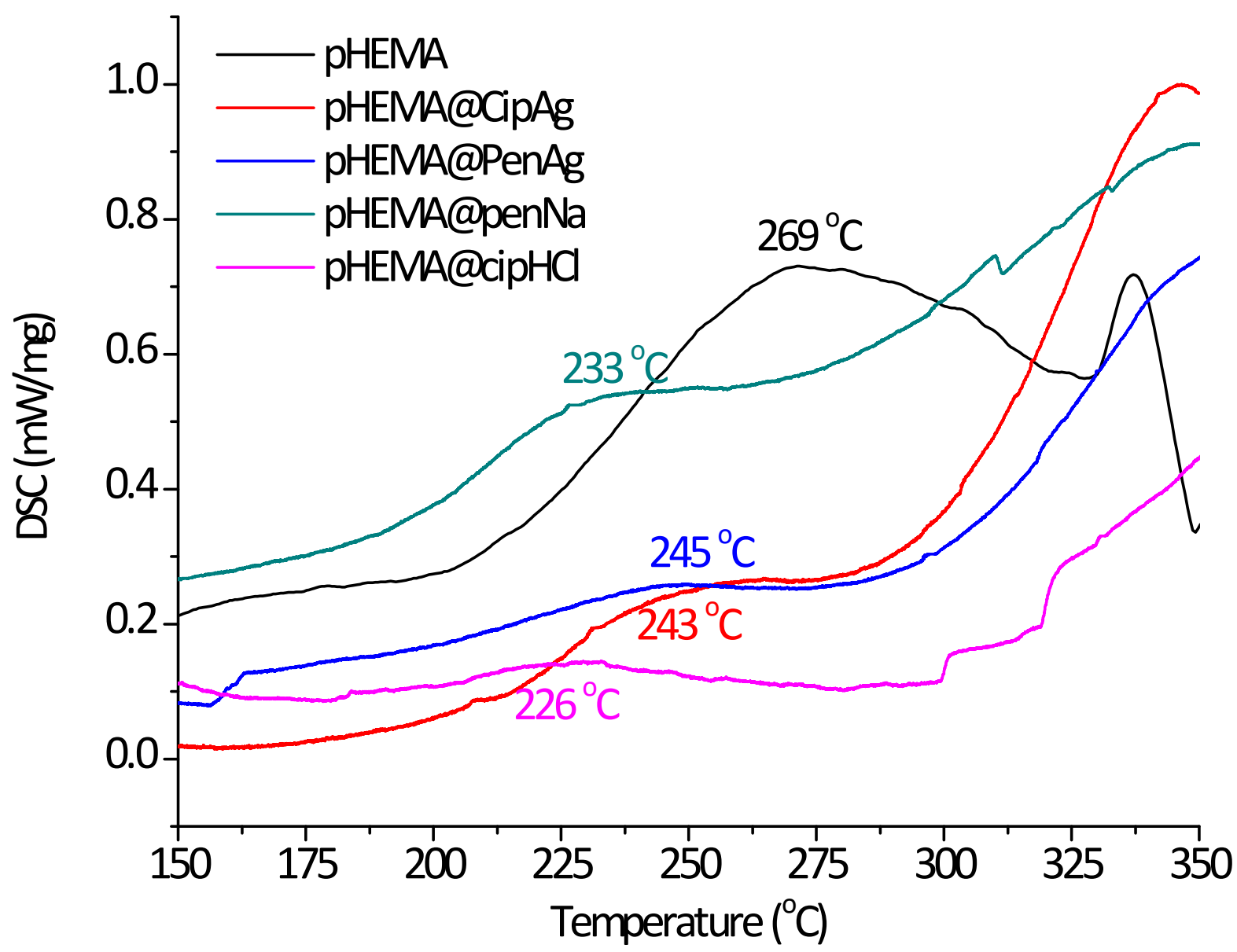

Figure S1. DSC diagrams on the powders of dry **pHEMA, pHEMA@CipAg, pHEMA@PenAg, pHEMA@{[CipH$_2$]$^+$Cl$^-$}, and pHEMA@PenNa.**
